# Preparative Production of Functionalized (*N*- and *O*-Heterocyclic) Polycyclic Aromatic Hydrocarbons by Human Cytochrome P450 3A4 in a Bioreactor

**DOI:** 10.3390/biom12020153

**Published:** 2022-01-18

**Authors:** Matic Srdič, Nico D. Fessner, Deniz Yildiz, Anton Glieder, Markus Spiertz, Ulrich Schwaneberg

**Affiliations:** 1 SeSaM-Biotech GmbH, 52074 Aachen, Germany; srdic@sesam-biotech.com; 2 Institute of Biotechnology, RWTH Aachen University, 52074 Aachen, Germany; 3 Institute of Molecular Biotechnology, Graz University of Technology, NAWI Graz, 8010 Graz, Austria; nico.fessner11@alumni.imperial.ac.uk; 4 DWI—Leibniz Institute for Interactive Materials, 52074 Aachen, Germany; yildiz@dwi.rwth-aachen.de; 5 Institute for Technical and Macromolecular Chemistry, RWTH Aachen University, 52074 Aachen, Germany; 6 Bisy GmbH, 8200 Hofstaetten an der Raab, Austria; anton.glieder@bisy.at

**Keywords:** cytochrome P450 3A4, CYP3A4, preparative-scale synthesis, PAHs, whole-cell biotransformation, bioreactor

## Abstract

Polycyclic aromatic hydrocarbons (PAHs) and their N- and O-containing derivatives (N-/O-PAHs) are environmental pollutants and synthetically attractive building blocks in pharmaceuticals. Functionalization of PAHs can be achieved via C-H activation by cytochrome P450 enzymes (e.g., P450 CYP3A4) in an environmentally friendly manner. Despite its broad substrate scope, the contribution of CYP3A4 to metabolize common PAHs in humans was found to be small. We recently showcased the potential of CYP3A4 in whole-cell biocatalysis with recombinant yeast *Komagataella phaffii* (*Pichia pastoris*) catalysts for the preparative-scale synthesis of naturally occurring metabolites in humans. In this study, we aimed at exploring the substrate scope of CYP3A4 towards (N-/O)-PAHs and conducted a bioconversion experiment at 10 L scale to validate the synthetic potential of CYP3A4 for the preparative-scale production of functionalized PAH metabolites. Hydroxylated products were purified and characterized using HPLC and NMR analysis. In total, 237 mg of fluorenol and 48 mg of fluorenone were produced from 498 mg of fluorene, with peak productivities of 27.7 μmol/L/h for fluorenol and 5.9 μmol/L/h for fluorenone; the latter confirmed that CYP3A4 is an excellent whole-cell biocatalyst for producing authentic human metabolites.

## 1. Introduction

Polycyclic aromatic hydrocarbons (PAHs) are environmental contaminants that are frequently formed during the combustion of organic material. PAHs can easily cross cell membranes through diffusion due to their lipophilic nature. However, although the parent PAH molecules themselves are not considered carcinogenic, their metabolites can form DNA ‘adducts’ leading to mutagenesis and cancer formation [1].

Conversely, PAH compounds are also building blocks in natural products [2] (e.g., fluorene [3,4], phenanthrene [5,6], anthracene [7,8], and phenanthridine [9,10]), possess molecular properties that qualify their application in optoelectronic devices (e.g., organic solar cells [11,12]), find applications in other technologies [13,14,15,16], or are subject of toxicological investigations. Their heterocyclic (*N*- and *O*-containing) derivatives (N-PAH/O-PAH) are especially attractive synthons in the pharmaceutical industries as part of active pharmaceutical ingredients (APIs) [17,18,19]. For all these applications, functionalization of the parent compounds is essential and can be achieved in late-stage C-H activation, for example, by cytochrome P450 enzymes (P450s) as an environmentally friendly alternative [20] to transition-metal bottom-up approaches [21,22].

Previous studies identified human liver P450 monooxygenases [23,24,25], such as CYP2A6, CYP2A13, and CYP1B1, as key enzymes responsible for the metabolic activation of PAHs in the human body [26,27,28]. Although the human CYP3A4 is renowned for its broad substrate scope and for metabolizing 50% of all FDA-approved drugs [25,29], its contribution towards PAH degradation was found to be comparatively small [27]. Hence, the metabolism of PAHs by P450 3A4 has not attracted much attention in the literature, despite the discovery that these compounds induced the CYP3A4 PXR promoter which enhances its expression [30].

Human P450s especially possess the potential to hydroxylate high-value lead compounds such as authentic human metabolites [31] or natural products [20,32,33]. However, low solubility due to the generally hydrophobic nature of PAHs was identified as a major limitation for efficient P450 biocatalysis when compared to chemical methods [34]. Synthesizing hundreds of milligrams of metabolites is often regarded as a synthetic challenge with human P450s due to their performance and requirement for co-factors [35,36,37,38]. Being able to scale-up production to achieve sufficient volumetric productivities and high yields with high oxygen transfer rates is therefore challenging too. The high redox requirements of monooxygenases were additionally reported as a major hindrance for their use at preparative scale [39]. Albeit not necessarily greener, the biocatalysis processes can be highly sustainable if renewable feedstocks are used in a resource-efficient manner [40].

Including stirred-tank cultures in the research process is important to facilitate later scale-up, as traditional shake-flask or microtiter plate cultures lack the same monitoring and control possibilities [41,42]. In addition, shaken cultures differ from stirred cultures in oxygen mass transfer, heat transfer, and agitation speed/type. Such scale-ups commonly cause problems, especially when using human CYPs due to their inherent instability [37,43], as well as strong dependency on the availability of the redox partner [35,38]. A key parameter that can be controlled in a bioreactor is dissolved oxygen that can be tweaked with temperature, aeration rate, headspace pressure, and stirring speed. This offers an advantage over shake-flask experiments for oxygen-transfer-rate-sensitive enzymes such as monooxygenases.

We recently showed that efficient expression levels of CYP3A4 in *Komagataella phaffii* (*Pichia pastoris*) enabled the preparative-scale synthesis of human testosterone metabolites in a bioreactor set-up [44] and observed activity of P450 3A4 towards selected PAH compounds [45]. Complementary to previous studies exploring the potential of CYP102A2 (BM3) [46,47,48,49,50] or other P450s to functionalize PAHs [45,51,52,53,54,55,56], this study evaluates P450 3A4′s substrate scope towards PAHs, N-PAHs, as well as O-PAHs. The goal was to preparatively synthesize authentic human metabolites under simple, reproducible conditions, in order to demonstrate the synthetic applicability and versatility of using this human P450. Metabolites of P450 3A4-catalyzed biotransformation of three PAHs were identified and an exemplary 10 L scale (6 L batch volume) bioreactor experiment enabled the conversion of 498 mg of fluorene (1) to produce 237 mg of 9-fluorenol (2) and 48 mg of fluorenone (3). Peak reactor volume productivity was calculated as 27.7 μmol/L/h for 2 and 5.9 μmol/L/h for 3.

## 2. Materials and Methods

All solvents and chemicals were purchased from SigmaAldrich/Merck (Steinheim/Darmstadt, Germany), VWR International (Fontenay-sous-Bois, France), Carl Roth GmbH (Karlsruhe, Germany) or InvivoGen (San Diego, CA, USA) in best available purity. HPLC gradient solvents were used for the HPLC measurements. HPLC tubes were bought from Macherey-Nagel (Düren, Germany) and the corresponding caps and inserts from Bruckner Analysentechnik (Linz, Austria) In experiments (2.1) an Agilent Technologies 1100 Series HPLC was used and in experiments (2.2) a Shimadzu Prominence LC-20AD HPLC was used. In both cases, separation was carried out via a Kinetex C18 (100 Å; 50 × 4.6 mm; 2.6 μm) reverse-phase column. The strirred-tank steel-jacket Bioengineering NLF22 10 L bioreactor from Bioengineering AG (Wald, Switzerland) was used for experiment (2.2). *K. phaffii* cells with expressed P450 3A4 were obtained from bisy GmbH (Hofstaetten, Austria) as lyophilized powder for experiments (2.1) and as biomass in carbon source exhausted basal salts medium [57] for experiment (2.2). OD measurements were executed with an Eppendorf BioPhotometer plus. NMR spectra were recorded on a Bruker Avance 400 spectrometer (operating at 400 MHz for ^1^H NMR and 101 MHz for ^13^C NMR) in CHCl^3^*-d* with tetramethylsilane (TMS) as internal standard purchased by Carl Roth.

### 2.1. Broad PAH Substrate Screening and HPLC

Whole-cell biotransformation for the substrate screening using commercial *K. phaffii* cells with co-expressed human P450 3A4 monooxygenase and human cytochrome P450 reductase (0.5 mM substrate in 400 μL, OD_600_ = 100, 28 °C, 320 rpm, 17 h) and the corresponding HPLC analysis (254 nm) were performed in the same way as reported before [45].

### 2.2. Bioreactor Biotransformations

The bioreactor biotransformation was performed using a commercial K. phaffii human P450 3A4 catalyst from 6 L of cell biomass at an OD_600_ of 200 with a dry cell weight that was measured as 90 g/L. Temperature was maintained at 28 °C and the pH at 7.4 with 20% ammonium solution and 20% phosphoric acid. Dissolved oxygen was maintained at 30% for the duration of the biotransformation, by changing stirring speed. Headspace pressure was maintained at 0.5 bar and aeration rate was 10 L/min. To initiate the biotransformation, 498 mg of fluorene was dissolved in 30 mL of hot (65 °C) methanol and added to 6 L of cell broth for a final concentration of 0.5 mM. Four 50 mL samples were taken at each time point to analyze the kinetics of the bioconversion. Time points were 0, 15, 30, 45, 60, 90, 120, 180, 300, 420, 540, 660, 1440, and 1560 min. Each sample was mixed with 50 mL of methanol to stop the biotransformation, vortexed and centrifuged (4000 g, 4 °C, 30 min), and 100 μL supernatant was taken for HPLC analysis. The percentage amounts of each compound in the samples were calculated with calibration curves from commercially bought reference compounds. The supernatants of samples at time points 0, 420 and, 1560 min were loaded in batches onto a C18 flash column (Chromabond^®^ Flash RS 40 C_18_ ec, 15–40 μm) obtained from Macherey-Nagel (Düren, Germany) for chromatographic purification using a Cytiva ÄKTA Pure 25 M (Marlborough, MA, US) device using the following method: Water containing 0.1% acetic acid (A) and methanol (B) was used for elution at room temperature (2 mL/min, 280 nm) in the following ratios: 0 mL: A/B 80/20; 120 mL: A/B 80/20; 200 mL: A/B 0/100; 330 mL A/B 0/100. The chromatographic fractions were pooled together for each time point and evaporated using a rotary evaporator. The wide mouth volumetric flask was then washed with (3 × 10 mL) EtOAc, transferred into a smaller volumetric flask, and evaporated under a stream of nitrogen. The obtained masses were off-white in color with a yellow fluorescent tint. The samples were weighed using a precision scale, then dissolved in deuterated chloroform for analysis using NMR.

### 2.3. NMR

NMR experiments were performed for the characterization and quantification of the biotransformation products. NMR spectra were recorded at room temperature for each compound and product mixture (20 mg/mL) that was initially purified as mentioned above. The following abbreviations were used throughout: s = singlet, d = doublet, t = triplet, q = quartet, sept. = septet, dd = doublet of doublet, etc., m = multiplet. Coupling constants (J) are given in Hz and refer to the given H,H-couplings.

9H-Fluorene (**1**, C_13_H_10_, white crystals, 212 mg, 42%): ^1^H NMR (400 MHz, Chloroform-*d*) δ = 7.80 (d, *J* = 7.5 Hz, 2H, H-4, H-5), 7.60–7.51 (m, 2H, H-1, H-8), 7.43–7.34 (m, 2H, H-2, H-7), 7.31 (td, *J* = 7.4, 1.2 Hz, 2H, H-3, H-6), 3.91 (s, 2H, H-9). ^13^C NMR (101 MHz, Chloroform-d) δ = 143.2 (C, C-8a, C-9a), 141.7 (C, C-4a, C-4b), 126.7 (CH, C-2, C-7), 126.7 (CH, C-3, C-6), 125.0 (CH, C-1, C-8), 119.9 (CH, C-4, C-5), 36.9 (CH2, C-9).

9-Flourenol (**2**, C_13_H_10_O, white crystals, 237 mg, 48%): ^1^H NMR (400 MHz, Chloroform-*d*) δ = 7.69–7.61 (m, 4H, H-1, H-4, H-5, H-8), 7.40 (td, *J* = 7.5, 1.2 Hz, 2H, H-2, H-7), 7.33 (td, *J* = 7.4, 1.1 Hz, 2H, H-3, H-6), 5.58 (s, 1H, H-9), 1.85 (s, 1H, -O*H*). ^13^C NMR (101 MHz, Chloroform-d) δ =145.7 (C, C-8a, C-9a), 140.0 (C, C-4a, C-4b), 129.1 (CH, C-2, C-7), 127.8 (CH, C-3, C-6), 125.1 (CH, C-1, C-8), 120.0 (CH, C-4, C-5), 75.3 (CH, C-9).

9-Fluorenone (**3**, C_13_H_8_O, yellow crystals, 48 mg, 10%): ^1^H NMR (400 MHz, Chloroform-*d*) δ = 7.68–7.61 (m, 2H, H-1, H-8), 7.53–7.43 (m, 4H, H-3, H-4, H-5, H-6), 7.28 (td, *J* = 7.2, 1.4 Hz, 2H, H-2, H-7). ^13^C NMR (101 MHz, Chloroform-d) δ = 193.9 (C, C-9), 144.4 (C, C-4a, C-4b), 134.7 (CH, C-3, C-6), 134.2 (C, C-8a, C-9a), 129.1 (CH, C-2, C-7), 124.3 (CH, C-1, C-8), 120.3 (CH, C-4, C-5).

## 3. Results and Discussion

### 3.1. Broad PAH Substrate Screening

**Figure 1:**
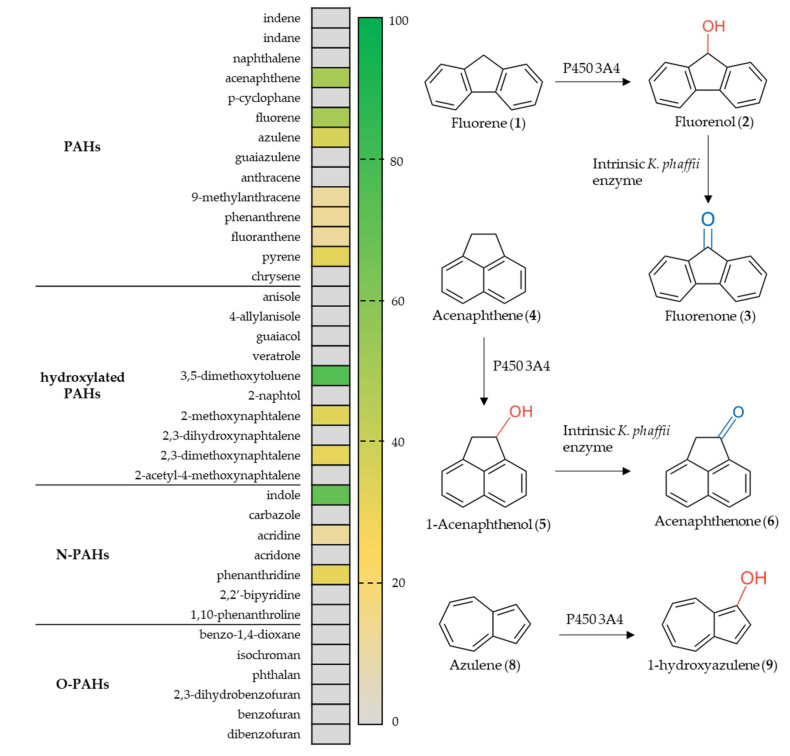
Screening of PAHs with CYP3A4. The heat map on the left indicates the conversion efficiency of P450 3A4 towards common PAHs, hydroxylated PAHs, as well as *N*- and *O*-containing PAHs. On the right, the synthetic fate of **1**, **4** and **8** upon *K. phaffii* whole-cell biotransformation employing CYP3A4 is depicted.

Firstly, the substrate scope of P450 3A4 towards PAHs, their *N*- and *O*-containing derivatives, as well as already hydroxylated PAH compounds were investigated (Figure 1). Notably, half of the 14 tested PAHs were accepted by P450 3A4 encompassing especially 3-cyclic, medium molecular weight (MMW) PAHs such as **1** (49% conversion; Figure S1) and acenaphthene (**4**, 51%; Figure S2). Authentic reference compounds allowed the identification of **2** and **3** as well as 1-hydroxyacenaphthene (**5**) and 1-acenaphthenone (**6**) as the products, respectively. Hence, in the latter case, we could confirm the literature results [28]. Interestingly, several new peaks were observed by HPLC for the conversion of 9-methylanthracene (**7**, 9%; Figure S3), whereas anthracene (0%) was not tolerated and phenanthrene (6%) only just. Apparently, the 9-methyl group of **7** is targeted by P450 3A4 as the activated benzylic position, as indicated by the appearance of a sharp, new peak. However, further smaller peaks were also observed, indicating either follow-up reactions with the product 9-hydroxymethylanthracene or that the 9-methyl group is required for active site crowding to allow the reaction [58]. None of the 2-cyclic, low-molecular-weight (LMW) PAHs such as indene or naphthalene were accepted, except for azulene (**8**, 35%; Figure S4), resulting in the formation of 1-hydroxyazulene (**9**) as indicated by an authentic reference. Higher molecular weight species (HMW) consisting of four fused cycles, such as fluoranthene (12%) and pyrene (**10**, 35%; Figure S5), were converted, as was to be expected by the renowned large active site cavity of P450 3A4 [59,60,61]. The conversion of **10** was particularly fascinating because at least five new product peaks could be observed, indicating a strong diversification of **10** by this human P450, which could be harnessed to produce several hydroxylated metabolites simultaneously. Generally, CYP3A4 seemed to accept mostly MMW and HMW species of PAHs, with azulene as the only LMW that was accepted.

In case of derivatives already bearing oxygen-containing substituents, some LMW PAHs were accepted. However, it seems likely that their communal methoxy group was dealkylated by P450 3A4, which is a common reaction of human liver P450s [62].

The conversion of the N-PAH substrates indole (68%), phenanthridine (**11**, 35%; Figure S6), and acridine (13; 5%) was in direct contrast to the observed activity towards non-heterocyclic containing equivalents of indene, phenanthrene, and anthracene. Therefore, the presence of the heterocyclic nitrogen atom within the heterocyclic compounds must be a prerequisite for being accepted by P450 3A4 as a substrate. The appearance of blue color suggested the formation of indigo from the electron-rich indole substrate. In addition, the presence of only one dominant product peak for the conversion of **11** implied the specific oxidation of one electron-rich position, possibly at *N*. Hence, we hypothesize *N*-oxide formation, which is a commonly occurring reaction for P450 3A4 [32].

Although O-PAHs are more electron-rich and thus more easily oxidized, the enzyme showed no discernible activity towards the selected O-PAH compounds. The lack of conversion of O-PAHs with low molecular weight could again be explained by the large CYP3A4 active site. In the case of dibenzofuran, it was further evidence for **2** being the product of the conversion of **1** because the *O*-heteroatom was occupying position 9.

### 3.2. Bioreactor Biotransformation

With the (N-/O)-PAH being accepted as substrates of P450 3A4, we aimed to scale up the biotransformation catalyzed by this *K. phaffi*-based whole-cell biocatalyst, from microtiter plates to a 10 L pilot scale fed-batch stirred-tank reactor, in order to show its potential for preparative purposes and thus tackle this highly restricting factor for the integration of biocatalysis in synthetic chemistry.

Compound **1** was selected as an exemplary substrate because it is a structural motif of many natural products [3,4], was hydroxylated with a relatively good percentage to **2** (49%), and was further oxidized to the corresponding ketone (**3**), allowing the illustration of a potential cascade reaction [63,64], which are important in biocatalysis as they enable more efficient synthesis routes by reducing the need for additional steps [34]. For the preparation of the CYP3A4 catalyst for this specific biotransformation, the most commonly cited *K. phaffii* fermentation buffer and cultivation conditions from the Invitrogen corporation *P. pastoris* fermentation guide [57] were used.

**Figure 2:**
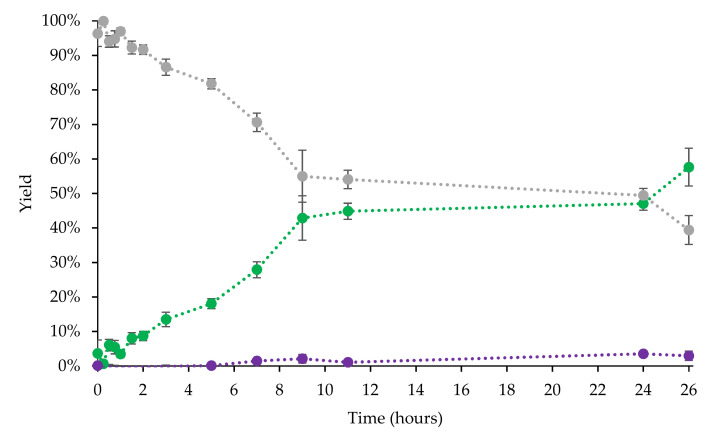
Bioreactor biotransformation conversion of **1** (grey dots) into **2** (green dots) and **3** (purple dots) as analyzed by HPLC. Percentage amounts in sample were determined using calibration curves obtained from commercially bought references.

The quantity of metabolites commonly used for in vitro and safety pharmacology testing are in the range of 0.1–100 mg [35], which we aimed to produce. We succeeded to isolate 237 mg of **2** and 48 mg of **3**, and also recovered 212 mg of **1**. To better characterize the kinetics and product diversification, we sampled a time series of 200 mL samples at the following time points (0 min, 15 min, 30 min, 45 min, 1 h, 1.5 h, 2 h, 3 h, 5 h, 7 h, 9 h, 11 h, 24 h, 26 h) for a total cultivation time of 26 h (Figure 2). All time point samples were extracted and analyzed by HPLC.

**Figure 3:**
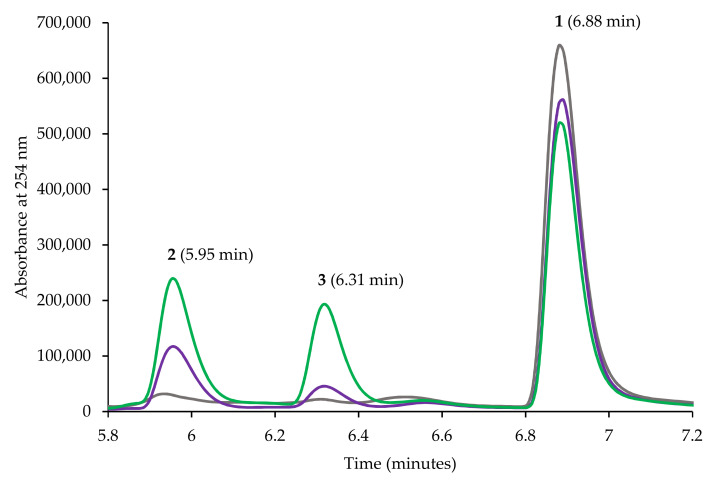
Bioreactor biotransformation conversion of **1** sample chromatograms at time points 0 h (grey line), 7 h (purple) and 26 h (green). Due to greater absorbance of **3** at 256 nm, the chromatographic peak is larger.

The conversion values for the points at 540 min, 660 min, and 1440 min were all approximately within the same error range, indicating that the conversion had slowed down considerably after the former time point. This is most likely due to the lack of glucose for intracellular NADPH regeneration. Other factors, such as high Km of the enzyme for this substrate, product inhibition or poor substrate partition coefficient due to its hydrophobic nature could also contribute to the slowdown. The high oxygen transfer rates achievable in a bioreactor could contribute to CYP3A4 degradation through H_2_O_2_ formation, especially if the cell chassis lacks the energy for reactive oxygen species disposal. The HPLC chromatograms for timepoints at 0, 420, and 1560 min are shown in Figure 3. The most abundant product formed was **2**, with a small amount of **3** present. The three molecules absorb differently at 254 nm, especially the ketone, likely due to the electron π- π * transition. Therefore, calibration curves were needed to assess the yield more accurately. We calculated the yields as 27.9% for **2** at 420 min and 57.6% at 1560 min. Yields for **3** were 1.5% at 420 min and 3% at 1560 min (Figure 3).

**Figure 4:**
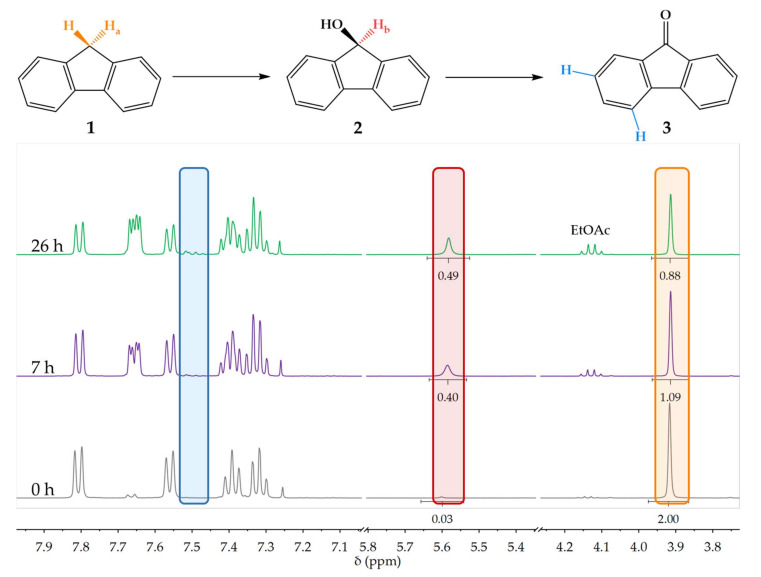
Stacked ^1^H NMR spectra at representative time intervals of the bioreactor biotransformation of sample **1** illustrate its conversion to **2** and **3** by the decrease in the aliphatic H-9 peak at 3.91 ppm (orange) and the simultaneous increase in the peak of the carbinol proton at 5.58 ppm (red), or the appearance of new peaks at 7.53–7.43 ppm (blue).

To confirm our results, we chromatographically purified samples taken at 0, 420, and 1560 min and analyzed their mol% yields using NMR, which is a more accurate method of quantifying PAHs [65]. The conversion could be monitored via the chemical shift of the hydrogen at 9-position (Figure 4). As **1** was converted (oxidized) into **2** and subsequently **3**, H-9 at 3.91 ppm (H_a_—orange highlight) first shifted downfield to 5.58 ppm and then disappeared. Therefore, the biotransformation of sample **1** into **2** and **3** could also be monitored by the decrease and increase in peak integration of the singlets at 3.91 ppm (H_a_) or 5.58 ppm (H_b_—red highlight), respectively. The formation of **3** could also be confirmed by the appearance of peaks at 7.48 ppm (blue highlight) that belong to aromatic protons of fluorenone in a new chemical environment. Maximal yield was 47.6 mol% of **2** and 9.7 mol% of **3** (Table 1).

It appears as if HPLC measurements overestimated the yield of **2** and underestimated the yield of **3**. Similar variance between analytical methods was reported before in the literature [65].

The most time efficient point was reached around 9 h after initiation. The long activity time showed that the yeast cells expressing CYP3A4 were indeed a stable biocatalyst with enzyme activity present, albeit diminished, even after 26 h.

Volume productivity (space–time yield) is a key sustainable catalysis metric [66]. After 7 h the bioreactor reaches its most productive stage, with productivities of 27.7 μmol/L/h for **2** and 5.9 μmol/L/h for **3**. With regard to sustainability, the reaction is inefficient after 9 h, when conversion leveled off, as shown in Figure 2. As a comparison, a very commonly cited biotransformation performed by CYP3A4 is the biotransformation of testosterone, where 6β-hydroxytestosterone is the major of multiple products [44]. Productivity numbers for the production of 6β-hydroxytestosterone were 6 μmol/L/h for CYP3A4 expressed in *E. coli* cells (100 g of wet cell mass, 1 L jacketed vessel, 48 h reaction time) [35,67], and 22 μmol/L/h for CYP3A4 expressed in *K. phaffii* cells (200 g of wet cell mass, 2 L jacketed vessel, 8 h reaction time) [44]. The productivity numbers we achieved in this study show that CYP3A4 is slightly worse at fluorene biotransformations compared to its model substrate, i.e., testosterone. In the second study, the total combined yield for all testosterone products was 70%; in the present study, we report a 57% combined conversion. A recently reported biotransformation of fluorene by CYP5035S7 from *Polyporus arcularius*, reported a combined yield for its two products, 2-hydroxyfluorene (35%) and 2,7-dihydroxyfluorene (18%), of 53% [45]. Although the two products formed are different, compared to CYP3A4, the total yield is similar.

During our broad PAH screening, we detected a 49% combined yield for **1**, meaning that scaling up the reaction from a 96-microtiter plate scale to a 10 L preparative scale improved yields slightly. It is likely that any of our screening reactions could be scaled-up in the same way and would improve volumetric productivity while maintaining the same yield. The frequently used media and bioreactor conditions we have chosen in this study matched the efficiency of microtiter plates, although could be further optimized. Oxygenation of the bioreactor could be increased drastically above 30% dissolved oxygen, by increasing aeration, headspace pressure, and agitation. Increased agitation would also assist in improving substrate mass transfer rates, as some CYP3A4 substrates, including PAHs, are quite hydrophobic and can form a biphasic layer. Supplementing the reaction buffer with a small amount of glucose or methanol could improve cofactor regeneration and extend the productivity of the reaction, which we investigated previously [44].

In total, we produced 237 mg of **2** and 48 mg of **3**. Although this yield is acceptable for the production of highly valuable pharmaceutical compounds, it is a concern that would need to be addressed in order to be economical in other industries [35]. For this to be achieved, efforts of both protein engineering, strain development, and bioprocessing will need to be combined.

## 4. Conclusions

In the present study we achieved the conversion of PAHs and their N-, O- and hydroxylated derivatives by the human P450 CYP3A4 expressed in *K. phaffi*, which proves that this system is a suitable whole-cell biocatalyst to produce oxidized PAHs in sufficient amounts for metabolic or pharmaceutical development. Several PAHs, hydroxylated PAHs, and N-PAHs were accepted as substrates, whereas none of the O-PAHs we selected were converted. Fluorene (**1**) was selected as the model substrate for the scale-up study because of its high yield, its positions as a structural motif in natural products, and the production of fluorenone (**3**) from fluorenol (**2**), which could illustrate a potential cascade reaction. Scale-up was performed in a 10 L jacketed steel bioreaction with 6 L of reaction broth volume using the most cited *K. phaffii* fermentation conditions from the Invitrogen *Pichia pastoris* fermentation guide. The employed cost-effective buffer systems will likely ensure a successful scale up of the developed PAHs products. Multiple samples were taken during the biotransformation to illustrate the kinetics of the reaction. All samples were analyzed using HPLC equipped with a UV–Vis detector and three representative time interval samples were analyzed using NMR. The final yield of **2** and **3** were 47.6% and 9.7%, respectively. Peak reactor volume productivity was reached after 7 h and was calculated as 27.7 μmol/L/h for **2** and 5.9 μmol/L/h for **3**. This productivity was sustained for up to 9 h of reaction time, after which it fell substantially. Total productivity after 26 h of reaction time was 9 μmol/L/h for **2** and 1.8 μmol/L/h for **3**. In total, we produced 237 mg of **2** and 48 mg of **3**. These results show that human CYP3A4 seems to participate in the metabolism of PAH compounds to a significant extent by accepting many of them and their derivatives as substrates. We demonstrated the possibility of scaling up the biotransformations of even such uncommon substrates for CYP3A4 to produce preparative scale product amounts for synthetic exploitation, the production of authentic human metabolites, or other valuable pharmaceutical compounds.

## Figures and Tables

**Table 1:** NMR-derived molar percentages of different time point samples.

	0 min (mol%)	420 min (mol%)	1560 min (mol%)
Fluorene (**1**)	97.1	52.9	42.7
Fluorenol (**2**)	2.9	38.8	47.6
Fluorenone (**3**)	0.0	8.3	9.7
Sampled weight in 200 mL	15.2 mg	14.7 mg	15.4 mg

## Data Availability

The data presented in this study are openly available for all figures and samples.

## Supplementary Materials

The following are available online at https://www.mdpi.com/article/10.3390/biom12020153/s1, Figures S1–S6; HPLC profiles of fluorene, acenaphthene, 9-methylanthracene, azulene, pyrene, phenanthridine respectively, catalyzed by CYP3A4. Figures S7 and S8; ^1^H and ^13^C NMR Spectrum of fluorene. Figures S9 and S10; ^1^H and ^13^C NMR Spectrum of fluorenol. Figures S11 and S12; ^1^H and ^13^C NMR Spectrum of fluorenone.

## References

[1] Moorthy B., Chu C., Carlin D.J. (2015). Polycyclic Aromatic Hydrocarbons: From Metabolism to Lung Cancer. Toxicol. Sci..

[2] Takikawa H., Nishii A., Sakai T., Suzuki K. (2018). Aryne-Based Strategy in the Total Synthesis of Naturally Occurring Polycyclic Compounds. Chem. Soc. Rev..

[3] Hussein E.M., Alsantali R.I., Abd El-Galil S.M., Obaid R.J., Alharbi A., Abourehab M.A.S., Ahmed S.A. (2019). Bioactive Fluorenes. Part I. Synthesis, Pharmacological Study and Molecular Docking of Novel Dihydrofolate Reductase Inhibitors Based-2,7-Dichlorofluorene. Heliyon.

[4] Shi Y., Gao S. (2016). Recent Advances of Synthesis of Fluorenone and Fluorene Containing Natural Products. Tetrahedron.

[5] Tóth B., Hohmann J., Vasas A. (2018). Phenanthrenes: A Promising Group of Plant Secondary Metabolites. J. Nat. Prod..

[6] Li S., Han L., Sun L., Zheng D., Liu J., Fu Y., Huang X., Wang Z. (2009). Synthesis and Antitumor Activities of Phenanthrene-Based Alkaloids. Molecules.

[7] Badria F.A., Ibrahim A.S. (2013). Evaluation of Natural Anthracene-Derived Compounds as Antimitotic Agents. Drug Discov. Ther..

[8] Debbab A., Aly A.H., Edrada-Ebel R., Wray V., Pretsch A., Pescitelli G., Kurtan T., Proksch P. (2012). New Anthracene Derivatives-Structure Elucidation and Antimicrobial Activity. Eur. J. Org. Chem..

[9] Lasák P., Motyka K., Kryštof V., Stýskala J. (2018). Synthesis, Bacteriostatic and Anticancer Activity of Novel Phenanthridines Structurally Similar to Benzo[c]Phenanthridine Alkaloids. Molecules.

[10] Donaldson L.R., Wallace S., Haigh D., Patton E.E., Hulme A.N. (2011). Rapid Synthesis and Zebrafish Evaluation of a Phenanthridine-Based Small Molecule Library. Org. Biomol. Chem..

[11] Aumaitre C., Morin J. (2019). Polycyclic Aromatic Hydrocarbons as Potential Building Blocks for Organic Solar Cells. Chem. Rec..

[12] Wang Y., Liu B., Koh C.W., Zhou X., Sun H., Yu J., Yang K., Wang H., Liao Q., Woo H.Y. (2019). Facile Synthesis of Polycyclic Aromatic Hydrocarbon (PAH)–Based Acceptors with Fine-Tuned Optoelectronic Properties: Toward Efficient Additive-Free Nonfullerene Organic Solar Cells. Adv. Energy Mater..

[13] Lo P.-Y., Lee G.-Y., Zheng J.-H., Huang J.-H., Cho E.-C., Lee K.-C. (2020). Intercalating Pyrene with Polypeptide as a Novel Self-Assembly Nano-Carrier for Colon Cancer Suppression In Vitro and In Vivo. Mater. Sci. Eng. C.

[14] Tümay S.O., Haddad Irani-nezhad M., Khataee A. (2020). Design of Novel Anthracene-Based Fluorescence Sensor for Sensitive and Selective Determination of Iron in Real Samples. J. Photochem. Photobiol. A Chem..

[15] Ziarani G.M., Moradi R., Lashgari N., Kruger H.G. (2018). Fluorene Dyes. Metal-Free Synthetic Organic Dyes.

[16] Van Damme J., Du Prez F. (2018). Anthracene-Containing Polymers toward High-End Applications. Prog. Polym. Sci..

[17] Cernak T., Dykstra K.D., Tyagarajan S., Vachal P., Krska S.W. (2016). The Medicinal Chemist’s Toolbox for Late Stage Functionalization of Drug-like Molecules. Chem. Soc. Rev..

[18] Vitaku E., Smith D.T., Njardarson J.T. (2014). Analysis of the Structural Diversity, Substitution Patterns, and Frequency of Nitrogen Heterocycles among U.S. FDA Approved Pharmaceuticals. J. Med. Chem..

[19] Singh P.K., Silakari O. (2018). The Current Status of O-Heterocycles: A Synthetic and Medicinal Overview. ChemMedChem.

[20] Fessner N.D. (2019). P450 Monooxygenases Enable Rapid Late-Stage Diversification of Natural Products via C−H Bond Activation. ChemCatChem.

[21] Liu J., Feng X. (2020). Bottom-Up Synthesis of Nitrogen-Doped Polycyclic Aromatic Hydrocarbons. Synlett.

[22] Hagui W., Doucet H., Soulé J.F. (2019). Application of Palladium-Catalyzed C(Sp2)–H Bond Arylation to the Synthesis of Polycyclic (Hetero)Aromatics. Chem.

[23] Guengerich F.P. (2017). Intersection of the Roles of Cytochrome P450 Enzymes with Xenobiotic and Endogenous Substrates: Relevance to Toxicity and Drug Interactions. Chem. Res. Toxicol..

[24] Rendic S., Guengerich F.P. (2012). Contributions of Human Enzymes in Carcinogen Metabolism. Chem. Res. Toxicol..

[25] Rendic S., Guengerich F.P. (2015). Survey of Human Oxidoreductases and Cytochrome P450 Enzymes Involved in the Metabolism of Xenobiotic and Natural Chemicals. Chem. Res. Toxicol..

[26] Shimada T., Murayama N., Kakimoto K., Takenaka S., Lim Y.-R., Yeom S., Kim D., Yamazaki H., Guengerich F.P., Komori M. (2017). Oxidation of 1-Chloropyrene by Human CYP1 Family and CYP2A Subfamily Cytochrome P450 Enzymes: Catalytic Roles of Two CYP1B1 and Five CYP2A13 Allelic Variants. Xenobiotica.

[27] Shimada T., Takenaka S., Kakimoto K., Murayama N., Lim Y.-R., Kim D., Foroozesh M.K., Yamazaki H., Guengerich F.P., Komori M. (2016). Structure–Function Studies of Naphthalene, Phenanthrene, Biphenyl, and Their Derivatives in Interaction with and Oxidation by Cytochromes P450 2A13 and 2A6. Chem. Res. Toxicol..

[28] Shimada T., Takenaka S., Murayama N., Yamazaki H., Kim J.-H., Kim D., Yoshimoto F.K., Guengerich F.P., Komori M. (2015). Oxidation of Acenaphthene and Acenaphthylene by Human Cytochrome P450 Enzymes. Chem. Res. Toxicol..

[29] Guengerich F.P. (2017). 2017 Human P450 Xenobiotics Toxicity. Chem. Res. Toxicol..

[30] Luckert C., Ehlers A., Buhrke T., Seidel A., Lampen A., Hessel S. (2013). Polycyclic Aromatic Hydrocarbons Stimulate Human CYP3A4 Promoter Activity via PXR. Toxicol. Lett..

[31] Winkler M., Geier M., Hanlon S.P., Nidetzky B., Glieder A. (2018). Human Enzymes for Organic Synthesis. Angew. Chem. Int. Ed..

[32] Fessner N.D., Grimm C., Srdic M., Weber H., Kroutil W., Schwaneberg U., Glieder A. (2021). Natural Product Diversification by One-Step Biocatalysis Using Human P450 3A4. ChemCatChem.

[33] Bernhardt R. (2006). Cytochromes P450 as Versatile Biocatalysts. J. Biotechnol..

[34] Dong J., Fernández-Fueyo E., Hollmann F., Paul C.E., Pesic M., Schmidt S., Wang Y., Younes S., Zhang W. (2018). Biocatalytic Oxidation Reactions: A Chemist’s Perspective. Angew. Chem. Int. Ed..

[35] Martinez C., Rupashinghe S. (2013). Cytochrome P450 Bioreactors in the Pharmaceutical Industry: Challenges and Opportunities. Curr. Top. Med. Chem..

[36] Zöllner A., Buchheit D., Meyer M.R., Maurer H.H., Peters F.T., Bureik M. (2010). Production of Human Phase 1 and 2 Metabolites by Whole-Cell Biotransformation with Recombinant Microbes. Bioanalysis.

[37] Urlacher V.B., Girhard M. (2019). Cytochrome P450 Monooxygenases in Biotechnology and Synthetic Biology. Trends Biotechnol..

[38] Bernhardt R., Urlacher V.B. (2014). Cytochromes P450 as Promising Catalysts for Biotechnological Application: Chances and Limitations. Appl. Microbiol. Biotechnol..

[39] Chakrabarty S., Wang Y., Perkins J.C., Narayan A.R.H. (2020). Scalable Biocatalytic C-H Oxyfunctionalization Reactions. Chem. Soc. Rev..

[40] Tieves F., Tonin F., Fernández-Fueyo E., Robbins J.M., Bommarius B., Bommarius A.S., Alcalde M., Hollmann F. (2019). Energising the E-Factor: The E+-Factor. Tetrahedron.

[41] Islam R.S., Tisi D., Levy M.S., Lye G.J. (2008). Scale-up of Escherichia Coli Growth and Recombinant Protein Expression Conditions from Microwell to Laboratory and Pilot Scale Based on Matched k La. Biotechnol. Bioeng..

[42] Marques M.P.C., Cabral J.M.S., Fernandes P. (2010). Bioprocess Scale-up: Quest for the Parameters to Be Used as Criterion to Move from Microreactors to Lab-Scale. J. Chem. Technol. Biotechnol..

[43] Geier M., Braun A., Emmerstorfer A., Pichler H., Glieder A. (2012). Production of Human Cytochrome P450 2D6 Drug Metabolites with Recombinant Microbes—A Comparative Study. Biotechnol. J..

[44] Fessner N.D., Srdič M., Weber H., Schmid C., Schönauer D., Schwaneberg U., Glieder A. (2020). Preparative-Scale Production of Testosterone Metabolites by Human Liver Cytochrome P450 Enzyme 3A4. Adv. Synth. Catal..

[45] Fessner N.D., Nelson D.R., Glieder A. (2021). Evolution and Enrichment of CYP5035 in Polyporales: Functionality of an Understudied P450 Family. Appl. Microbiol. Biotechnol..

[46] De Almeida Santos G., Dhoke G.V., Davari M.D., Ruff A.J., Schwaneberg U. (2019). Directed Evolution of P450 BM3 towards Functionalization of Aromatic O-Heterocycles. Int. J. Mol. Sci..

[47] Sideri A., Goyal A., Di Nardo G., Tsotsou G.E., Gilardi G. (2013). Hydroxylation of Non-Substituted Polycyclic Aromatic Hydrocarbons by Cytochrome P450 BM3 Engineered by Directed Evolution. J. Inorg. Biochem..

[48] Sulistyaningdyah W.T., Ogawa J., Li Q.-S., Maeda C., Yano Y., Schmid R.D., Shimizu S. (2005). Hydroxylation Activity of P450 BM-3 Mutant F87V towards Aromatic Compounds and Its Application to the Synthesis of Hydroquinone Derivatives from Phenolic Compounds. Appl. Microbiol. Biotechnol..

[49] Li Q.-S., Ogawa J., Schmid R.D., Shimizu S. (2001). Engineering Cytochrome P450 BM-3 for Oxidation of Polycyclic Aromatic Hydrocarbons. Appl. Environ. Microbiol..

[50] Urlacher V., Schmid R.D. (2002). Biotransformations Using Prokaryotic P450 Monooxygenases. Curr. Opin. Biotechnol..

[51] Syed K., Porollo A., Miller D., Yadav J.S. (2013). Rational Engineering of the Fungal P450 Monooxygenase CYP5136A3 to Improve Its Oxidizing Activity toward Polycyclic Aromatic Hydrocarbons. Protein Eng. Des. Sel..

[52] Syed K., Porollo A., Lam Y.W., Grimmett P.E., Yadav J.S. (2013). CYP63A2, a Catalytically Versatile Fungal P450 Monooxygenase Capable of Oxidizing Higher-Molecular-Weight Polycyclic Aromatic Hydrocarbons, Alkylphenols, and Alkanes. Appl. Environ. Microbiol..

[53] Syed K., Porollo A., Lam Y.W., Yadav J.S. (2011). A Fungal P450 (CYP5136A3) Capable of Oxidizing Polycyclic Aromatic Hydrocarbons and Endocrine Disrupting Alkylphenols: Role of Trp129 and Leu324. PLoS ONE.

[54] Syed K., Doddapaneni H., Subramanian V., Lam Y.W., Yadav J.S. (2010). Genome-to-Function Characterization of Novel Fungal P450 Monooxygenases Oxidizing Polycyclic Aromatic Hydrocarbons (PAHs). Biochem. Biophys. Res. Commun..

[55] Torres E., Hayen H., Niemeyer C.M. (2007). Evaluation of Cytochrome P450BSβ Reactivity against Polycyclic Aromatic Hydrocarbons and Drugs. Biochem. Biophys. Res. Commun..

[56] Fessner N.D., Grimm C., Kroutil W., Glieder A. (2021). Late-Stage Functionalisation of Polycyclic (N-Hetero-) Aromatic Hydrocarbons by Detoxifying CYP5035S7 Monooxygenase of the White-Rot Fungus Polyporus Arcularius. Biomolecules.

[57] (2021). Invitrogen. *Pichia Fermentation Process Guidelines Overview*; Invitrogen: 2002. http://tools.thermofisher.com/content/sfs/manuals/pichiaferm_prot.pdf.

[58] Schiavini P., Cheong K.J., Moitessier N., Auclair K. (2017). Active Site Crowding of Cytochrome P450 3A4 as a Strategy To Alter Its Selectivity. ChemBioChem.

[59] Hackett J.C. (2018). Membrane-Embedded Substrate Recognition by Cytochrome P450 3A4. J. Biol. Chem..

[60] Ekroos M., Sjögren T. (2006). Structural Basis for Ligand Promiscuity in Cytochrome P450 3A4. Proc. Natl. Acad. Sci. USA.

[61] Paloncýová M., Navrátilová V., Berka K., Laio A., Otyepka M. (2016). Role of Enzyme Flexibility in Ligand Access and Egress to Active Site: Bias-Exchange Metadynamics Study of 1,3,7-Trimethyluric Acid in Cytochrome P450 3A4. J. Chem. Theory Comput..

[62] Donato M.T., Jiménez N., Castell J.V., Gómez-Lechón M.J. (2004). Fluorescence-Based Assays for Screening Nine Cytochrome P450 (P450) Activities in Intact Cells Expressing Individual Human P450 Enzymes. Drug Metab. Dispos..

[63] Rudroff F. (2019). Whole-Cell Based Synthetic Enzyme Cascades—Light and Shadow of a Promising Technology. Curr. Opin. Chem. Biol..

[64] Wu S., Li Z. (2018). Whole-Cell Cascade Biotransformations for One-Pot Multistep Organic Synthesis. ChemCatChem.

[65] Weißhoff H., Preiß A., Nehls I., Win T., Mügge C. (2002). Development of an HPLC-NMR Method for the Determination of PAHs in Soil Samples—A Comparison with Conventional Methods. Anal. Bioanal. Chem..

[66] Lange J.-P. (2021). Performance Metrics for Sustainable Catalysis in Industry. Nat. Catal..

[67] Vail R.B., Homann M.J., Hanna I., Zaks A. (2005). Preparative Synthesis of Drug Metabolites Using Human Cytochrome P450s 3A4, 2C9 and 1A2 with NADPH-P450 Reductase Expressed in Escherichia Coli. J. Ind. Microbiol. Biotechnol..

