# Supplementary material for: Preparative Production of Functionalized (N- and O-Heterocyclic) Polycyclic Aromatic Hydrocarbons by Human Cytochrome P450 3A4 in a Bioreactor
Source: Biomolecules. 2022 Jan 18;12(2):153. doi: 10.3390/biom12020153 (PMC8961652; doi:10.3390/biom12020153)
Supplement: Supplementary file 1 [file biomolecules-12-00153-s001.zip › biomolecules-1541162-supplementary.pdf]

Biomolecules

Supporting Information

for

Preparative Production of Functionalized (*N*- and *O*-Heterocyclic) Polycyclic Aromatic Hydrocarbons by Human Cytochrome P450 Enzyme 3A4 in a Bioreactor

Matic Srdič [1,2], Nico D. Fessner [3], Deniz Yildiz [4,5], Anton Glieder [6], Markus Spiertz [1*], Ulrich Schwaneberg [2,4,*]

1 SeSaM-Biotech GmbH, Aachen, Germany; srdic@sesam-biotech.com (M.Sr.), spiertz@sesam-biotech.com (M.Sp.)
2 Institute of Biotechnology, RWTH Aachen University, Aachen, Germany; u.schwaneberg@biotec.rwth-aachen.de (U.S.)
3 Institute of Molecular Biotechnology, Graz University of Technology, NAWI Graz, Graz, Austria; nico.fessner11@alumni.imperial.ac.uk (N.D.F), a.glieder@tugraz.at (A.G.)
4 DWI—Leibniz Institute for Interactive Materials, Aachen, Germany; yildiz@dwi.rwth-aachen.de (D.Y.)
5 Institute for Technical and Macromolecular Chemistry, RWTH Aachen University, Aachen, Germany.
6 Bisy GmbH, Hofstaetten/Raab, Austria; anton.glieder@bisy.at (A.G.)

* Correspondence: spiertz@sesam-biotech.com; u.schwaneberg@biotec.rwth-aachen.de

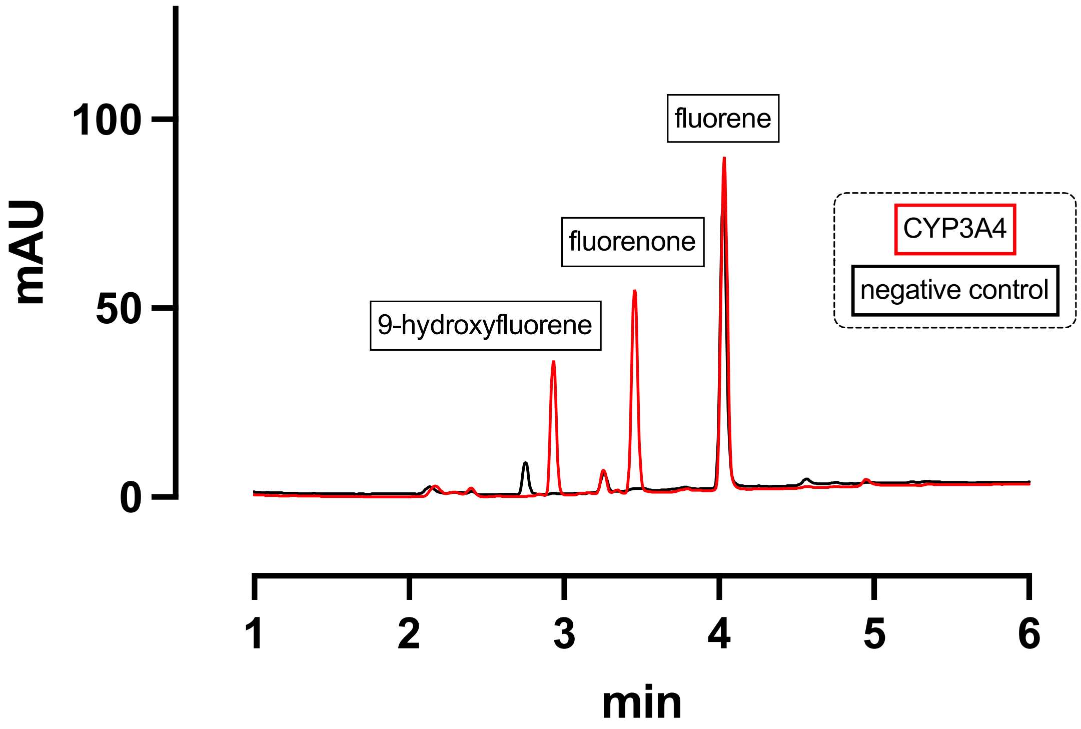

Figure S1: HPLC profile of fluorene conversion catalysed by CYP3A4.

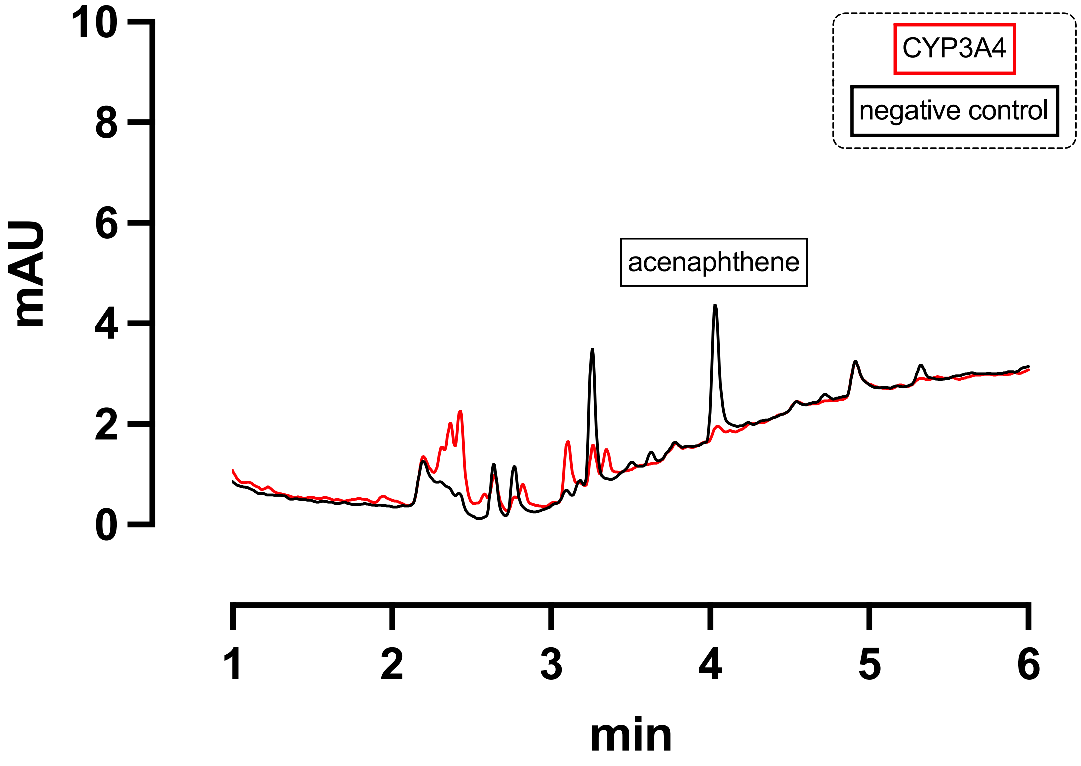

Figure S2: HPLC profile of acenaphthene conversion catalysed by CYP3A4.

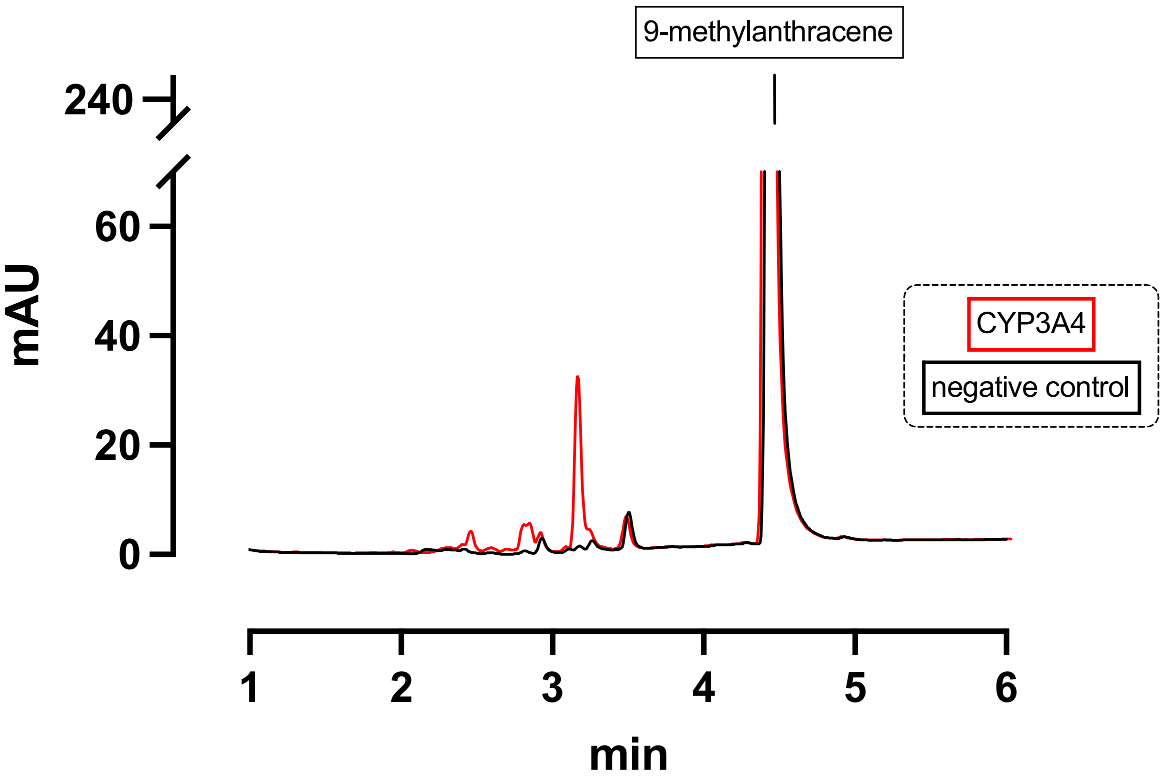

Figure S3: HPLC profile of 9-methylanthracene conversion catalysed by CYP3A4.

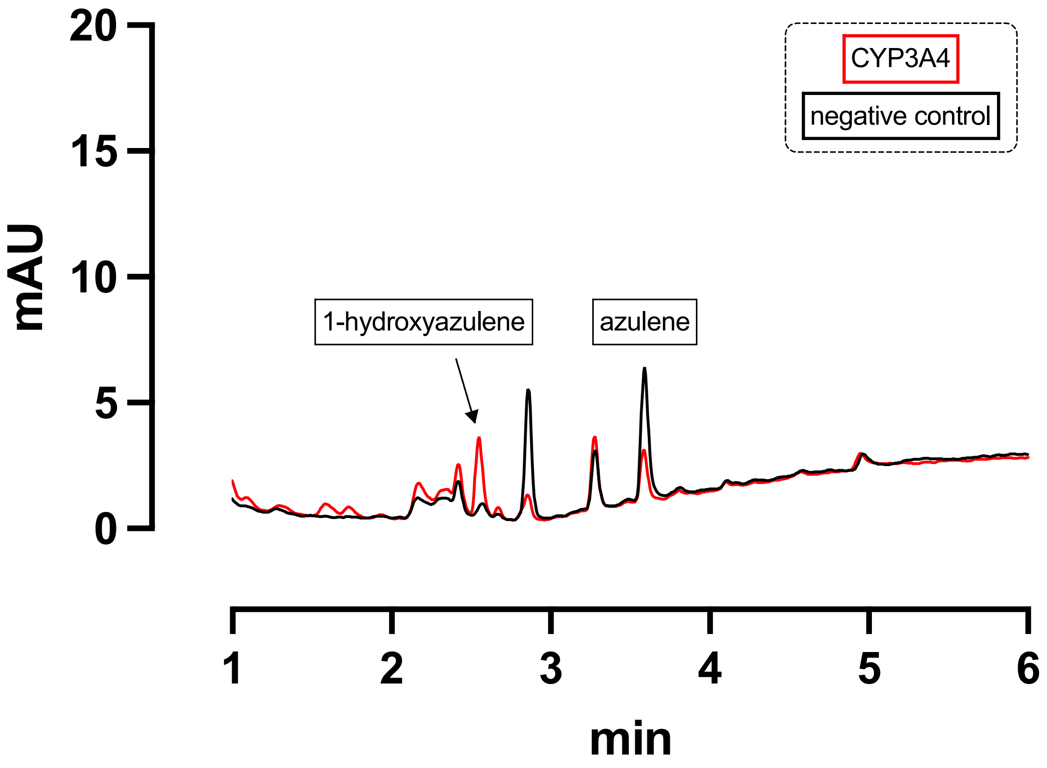

Figure S4: HPLC profile of azulene conversion catalysed by CYP3A4.

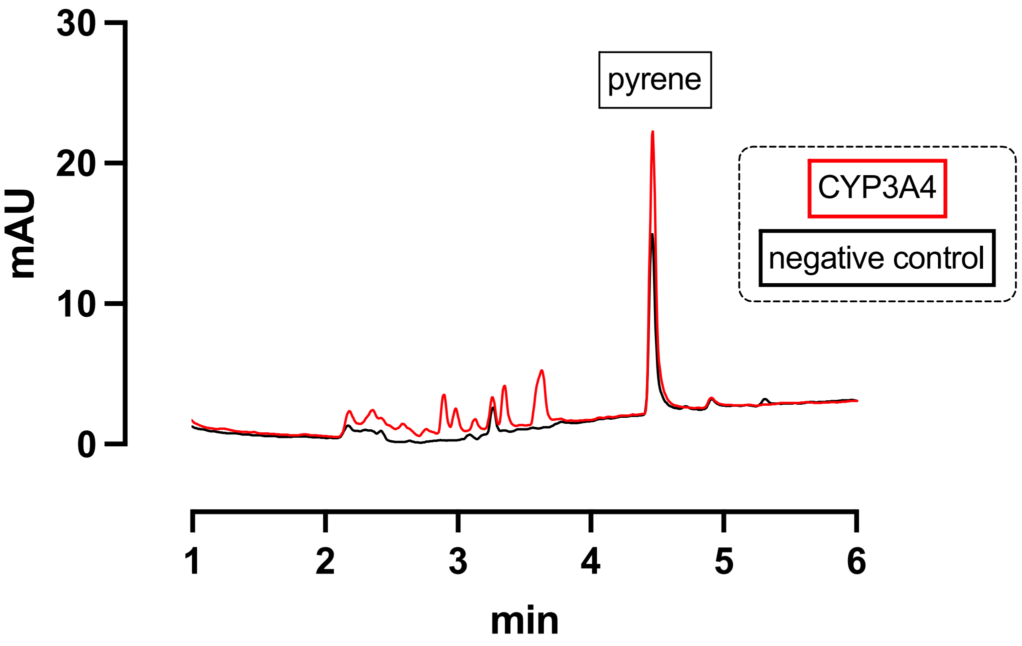

Figure S5: HPLC profile of pyrene conversion catalysed by CYP3A4.

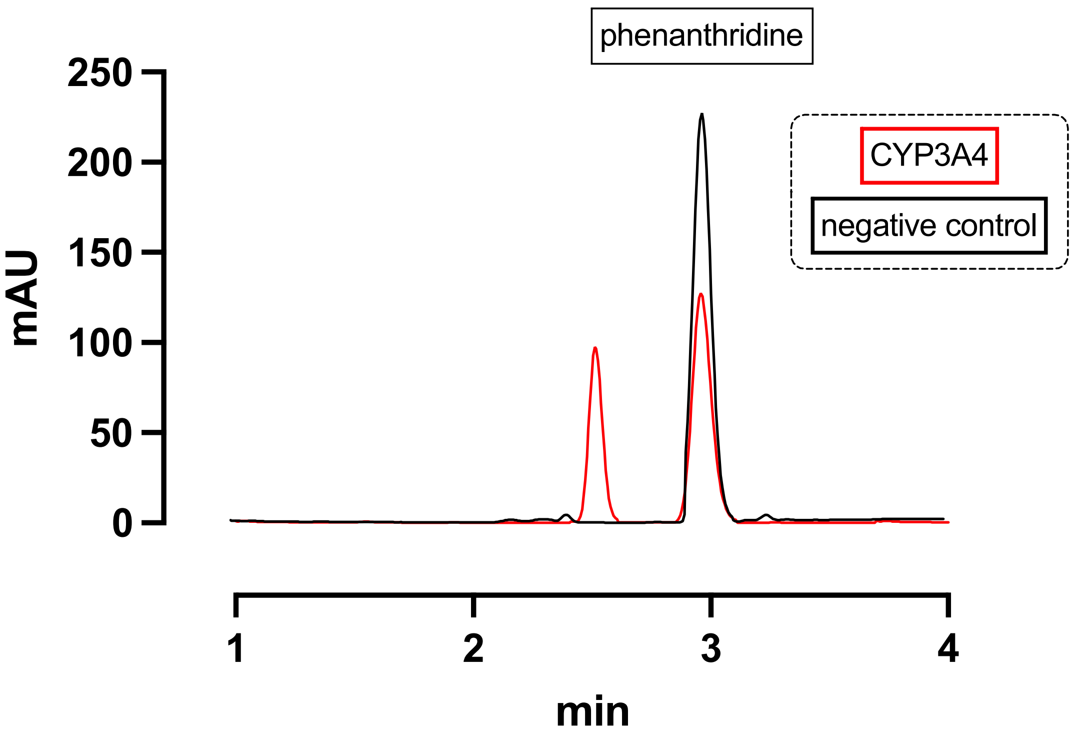

Figure S6: HPLC profile of phenanthridine conversion catalysed by CYP3A4.

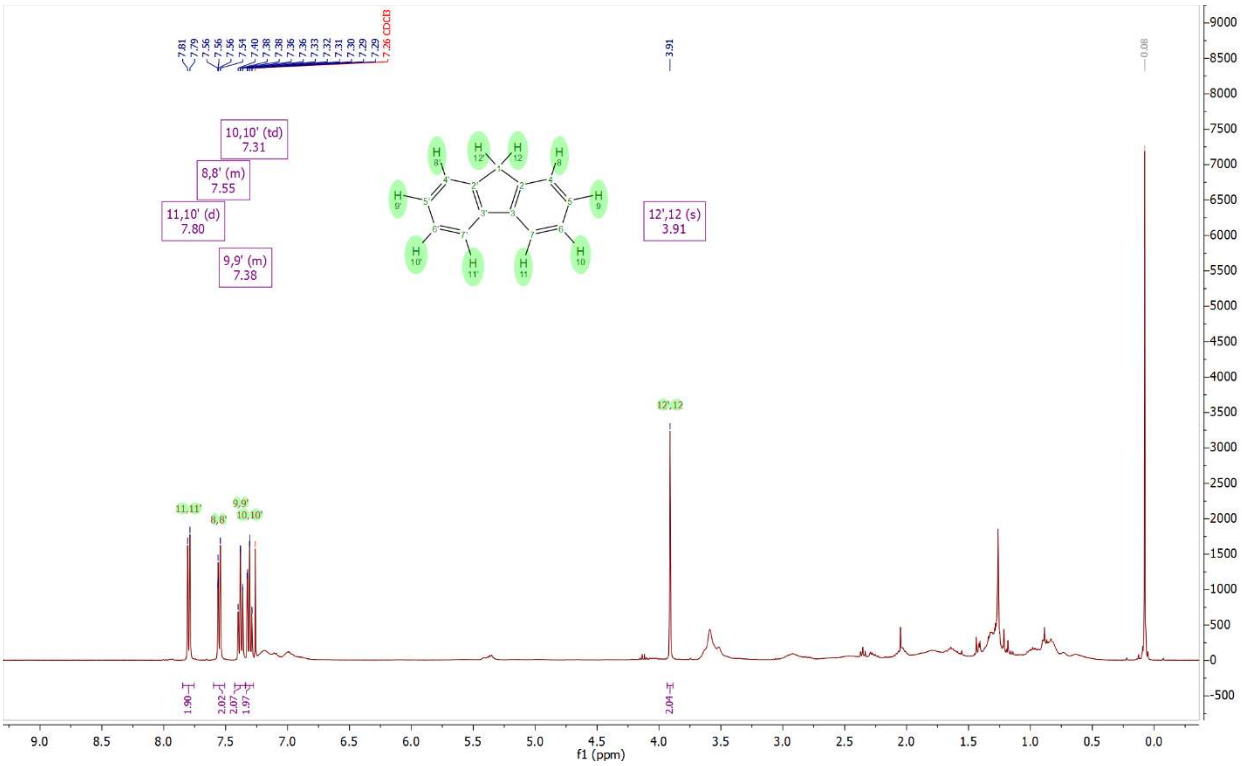

Figure S7: ^{1}H NMR Spectrum of 9*H*-Fluorene.

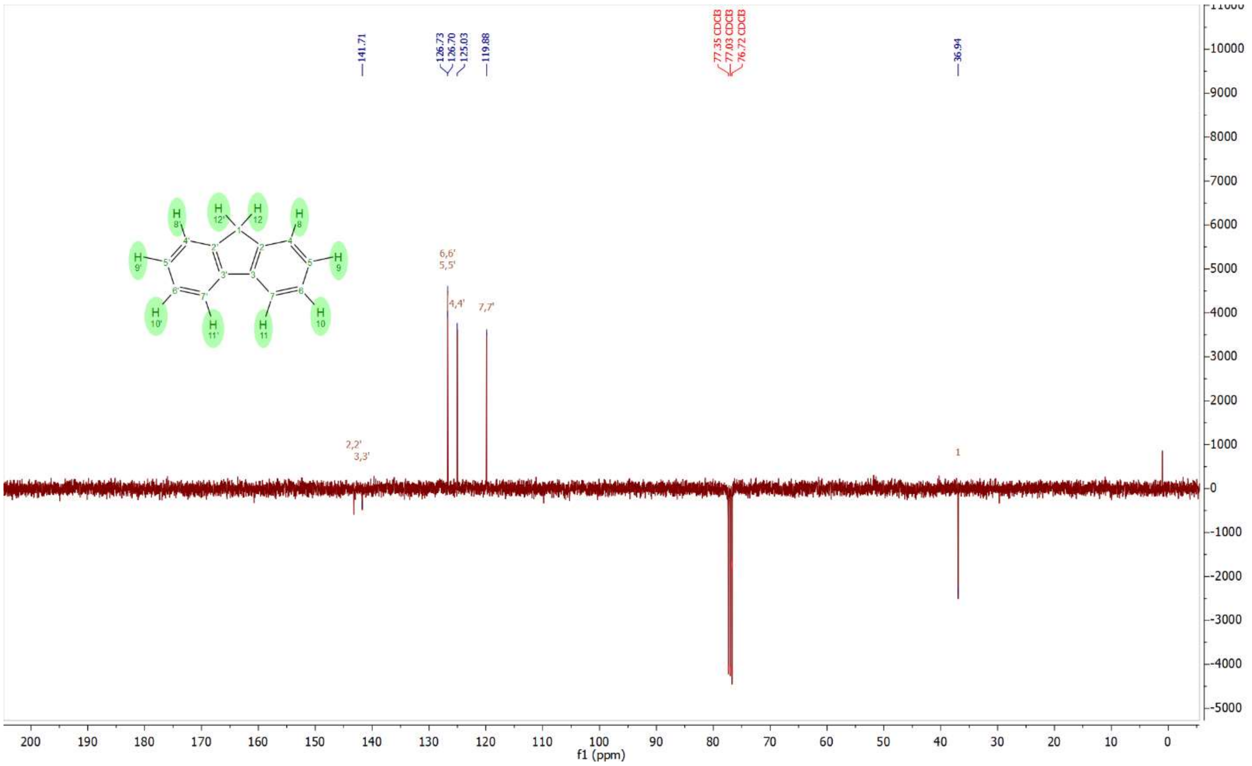

Figure S8: ^{13}C NMR Spectrum of 9*H*-Fluorene.

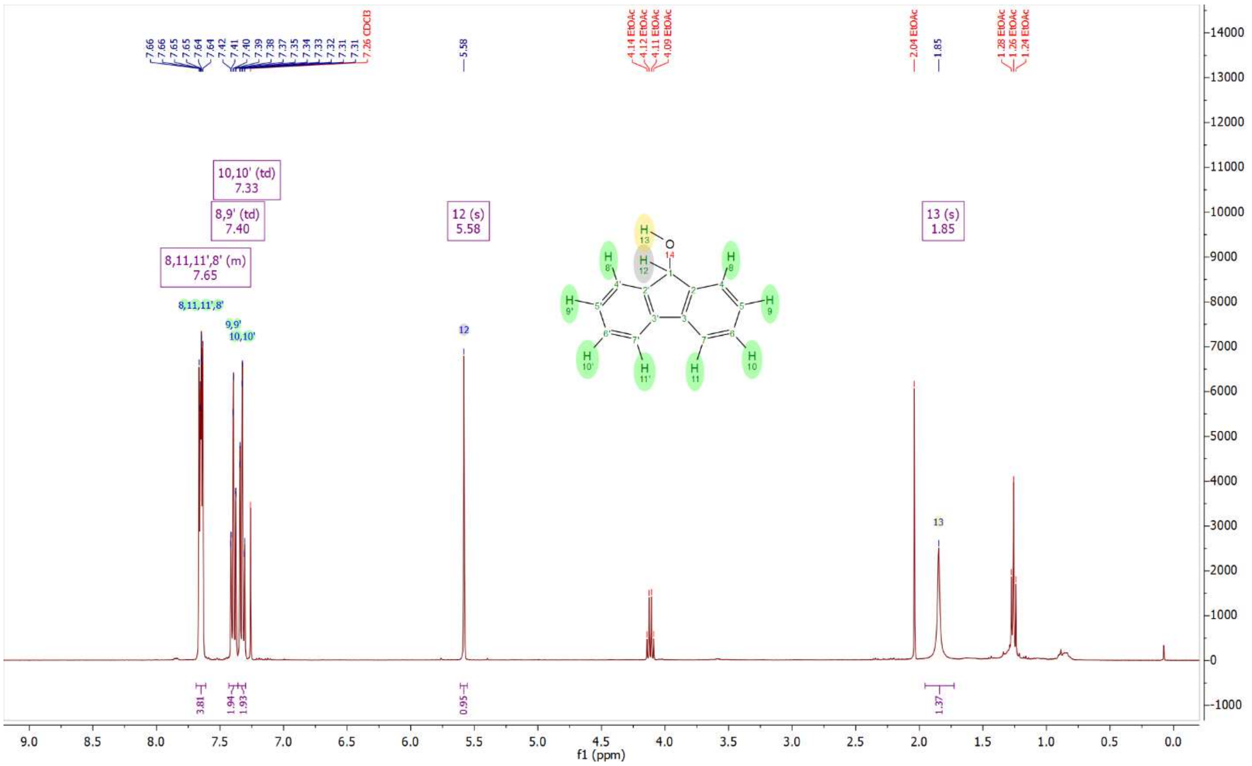

Figure S9: ^{1}H NMR Spectrum of 9-hydroxyfluorene.

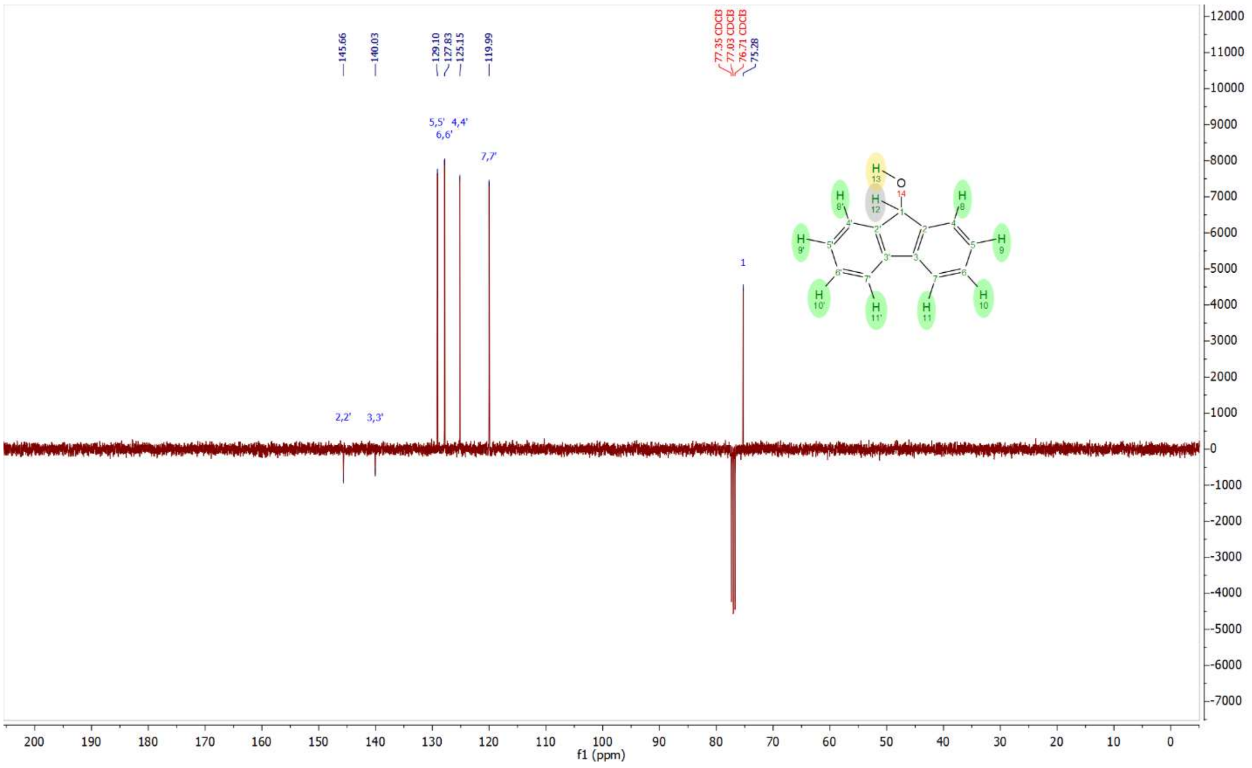

Figure S10: ^{13}C NMR Spectrum of 9-hydroxyfluorene.

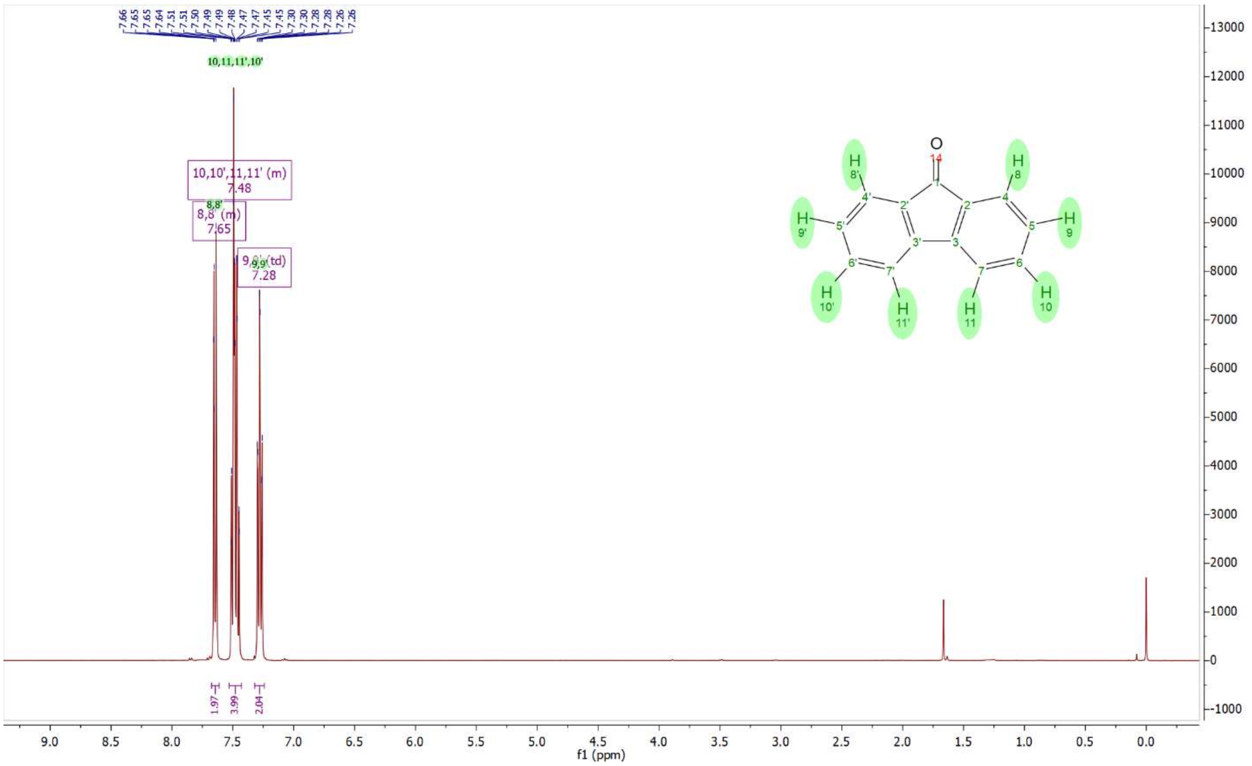

Figure S11: ^{1}H NMR Spectrum of 9-Fluorenone.

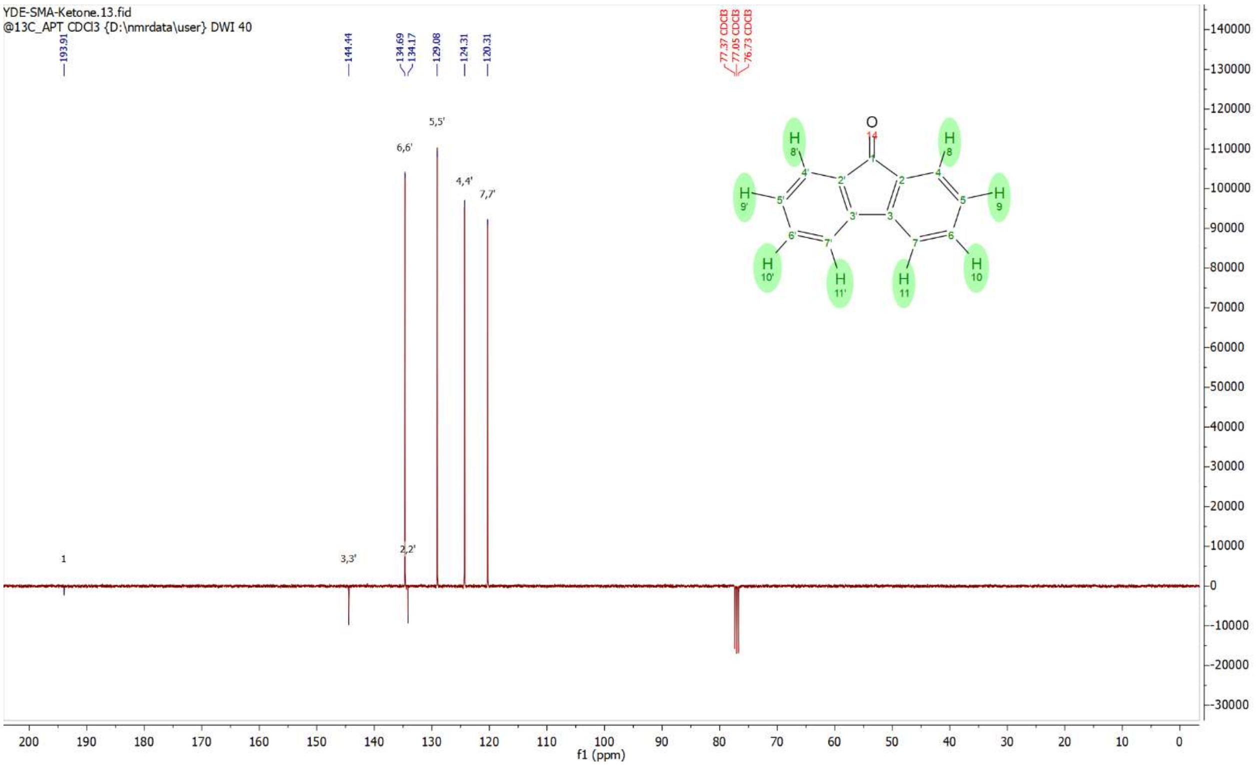

Figure S12: ^{13}C NMR Spectrum of 9-Fluorenone.
